# Dying too soon or living too long? Withdrawing treatment from patients with prolonged disorders of consciousness after *Re Y*

**DOI:** 10.1186/s12910-019-0424-4

**Published:** 2019-12-30

**Authors:** Richard Huxtable

**Affiliations:** Centre for Ethics in Medicine, Population Health Sciences, Bristol Medical School, Canynge Hall, 39 Whatley Road, Bristol, BS8 2PS UK

**Keywords:** Best interests, Minimally conscious state, Prolonged disorders of consciousness, Vegetative state

## Abstract

**Background:**

In the ruling in *Y* [2018], the UK Supreme Court has confirmed that there is no general requirement for the courts in England and Wales to authorise the withdrawal of clinically assisted nutrition and hydration from patients with prolonged disorders of consciousness. The perceived requirement, which originated in a court ruling in 1993, encompassed those in the vegetative state and those in the minimally conscious state. The ruling in *Y* confirms that the court may still be approached to decide difficult or contested cases, but there is otherwise no routine requirement that the judges be approached.

**Main body:**

There is much to welcome in this ruling, particularly as it means that these decisions for these patients are no longer (unusually) singled out for a judicial decision, with all the financial and emotional costs that court proceedings can entail. However, there is also a risk that the ruling might have unwelcome consequences. First, there is the possibility that patients might die too soon, particularly if doctors should now adopt the courts’ previous reasoning, which has suggested that patients in the vegetative state lack interests, so treatment may – perhaps must – be withdrawn. Secondly, there is the converse possibility that patients might live too long, since empirical research suggests that – whether intentionally or not – patients’ families, clinicians, and the health system appear to promote treatment-by-default.

**Conclusion:**

Rather than adopt general positions, which may be contestable and potentially risky, this article argues, on a pluralistic basis, that the individual patient should be the focus of any decision made in his or her ‘best interests’. The existing legal framework in England and Wales, which is provided by the Mental Capacity Act 2005, already points in this direction, although more efforts may be needed to ensure that those involved in making these decisions are suitably educated and supported. Fortunately, new guidance from the British Medical Association could help clinicians and families to make decisions in the future, which are appropriate for the incapacitated individual patient in question.

## Background

English law has long required – or, at least, has appeared to require – that decisions to withdraw clinically assisted nutrition and hydration (CANH) from patients with prolonged disorders of consciousness (PDOC) be made by a court. A PDOC ‘refers to a state where a patient has wakefulness but absent or reduced awareness for more than 4 weeks’ ([1], p. 1). The term encompasses both those in the ‘permanent vegetative state’ (PVS), who have no awareness, and those in the ‘minimally conscious state’ (MCS), who have reduced awareness [2]. CANH refers to feeding by tube, rather than orally, and therefore to the provision of nutrition and hydration via (for example) nasogastric tube, percutaneous endoscopic gastrostomy or parenteral nutrition ([3], p. 6).

The (apparent) requirement to come to court arose from the first ruling in this jurisdiction to consider such a decision for a patient in the ‘persistent vegetative state’, as it was then called. In *Bland* in 1993, the then highest English court held that such treatment was not in the best interests of the patient, Anthony Bland, so it could be withdrawn [4]. Recognising that the court was in unfamiliar, controversial territory, Lord Goff endorsed the position of the lower courts: as a matter of practice, ‘the opinion of the court should be sought in all cases similar to the present’, ‘at least for the time being and until a body of experience and practice has been built up which might obviate the need for application in every case’ ([4], p. 873). Lord Goff hoped, however, that the President of the High Court ‘will soon feel able to relax the present requirement so as to limit applications for declarations to those cases in which there is a special need for the procedure to be invoked’ ([4], p. 874).

In 2007, the British Medical Association also hoped the ‘requirement’ would soon be relaxed ([5], p. 61), but it persisted and, that year, was extended to encompass the withdrawal of CANH from patients in the MCS [6]. At this time, the Mental Capacity Act 2005 came into force, with the effect that the new Court of Protection would now deal with these cases. The Act governs the (non-)treatment of adults who lack capacity, empowering adults to create an ‘advance decision to refuse treatment’ or confer a ‘lasting power of attorney’ in advance of losing capacity ([7], sections 9–14, 24–26). The latter, however, does not confer an unfettered authority on its donee, since the donee’s decision must align with the ‘best interests’ of the patient ([7], section 9(4)). Moreover, this is generally the test to be applied if the patient has not created an advance decision to refuse treatment i.e. decisions must be made in the ‘best interests’ of the incapacitated individual ([7], section 4). The new Court would not routinely need to make these decisions; provided there was no disagreement, decisions would usually be led by the responsible clinician, albeit in consultation with those close to the patient. However, along with the Act came Practice Direction 9E which held that withholding or withdrawing CANH from patients in PDOC ‘should be regarded as serious medical treatment … and should be brought to the court’ ([6], para. 5).

The situation began to change in 2017. The Court of Protection Rules Committee withdrew Practice Direction 9E, with effect from 1 December 2017, and case law cast further doubt on the ‘requirement’ [8–10]. These cases culminated, in July 2018, with a ruling from the (now) highest court, the Supreme Court, in *Y* [2018], which decisively does away with the requirement for a court to make decisions about CANH for patients in the PVS or MCS, at least where the case is clear and uncontested [11].

In their analysis of the demise of the Liverpool Care Pathway [12], Seymour and Clark have called for ‘greater assessment of the wider risks and more careful consideration of the unintended consequences that might result from the roll out of new end-of-life interventions’ [13]. *Y* might be considered such an intervention and certainly invites such an assessment. On one view, this is a welcome ruling, likely to have positive effects. In this article, however, I also query whether the decision might also have unwelcome consequences and I offer some suggestions for avoiding or ameliorating these. I have two – opposing – consequences in view: the first is that patients might die too soon, and the second is that patients might live too long. My argument is inter-disciplinary, since it is primarily ethical, whilst also drawing on legal and social scientific sources. In the UK, social scientific research in this area has been substantially led by the Coma and Disorders of Consciousness Research Centre [14], whose members – in particular Jenny and Celia Kitzinger – have undertaken a range of theoretical and empirical research, on which I will draw. Theirs are qualitative (not quantitative) data, from which, some argue, it is inappropriate to generalise [15]. However, the broader commonality of their findings and themes indicate that there may be a case for their generalisability (or, at least, transferability) [16], particularly when combined with ethical arguments as I seek to do here.^1^

## Main body

### The decision in *Y* [2018]

Briefly summarised, the Supreme Court decision concerned Mr. Y, who had suffered a cardiac arrest in June 2017, which resulted in severe cerebral hypoxia and extensive brain damage. He did not recover consciousness after the arrest. In September, his treating clinician concluded he had a PDOC; in October, an expert second opinion considered him to be in a vegetative state (VS). The clinical team and Y’s family agreed that CANH should be withdrawn. Court proceedings began in November, specifically to confirm that court approval was not mandatory and that no civil or criminal liability would result if CANH were withdrawn. The Queen’s Bench Division agreed, but a direct appeal to the Supreme Court was permitted [18]. Mr. Y died in December, with CANH in place, after having developed acute respiratory sepsis, but the appeal proceeded given the importance of the issues.

The Supreme Court dismissed the appeal. Delivering the court’s judgment, Lady Black held that neither domestic law nor the European Convention on Human Rights (ECHR) gave rise to a mandatory requirement to involve the court in making decisions about withdrawing CANH from every patient with a PDOC. The fundamental question for a doctor or a court was whether it was lawful to provide treatment. Leaving aside any advance decision to refuse treatment or lasting power of attorney ([7], sections 9–14, 24–26), lawfulness turned on the best interests of the patient. The common law did not impose a legal requirement to come to court for these best interests decisions for these patients. *Bland* made only a good practice recommendation [4]. The 2005 Act also did not impose any such requirement (indeed, although the Act adopted many of the Law Commission’s preceding recommendations, it did not enact this particular recommendation). The accompanying Code of Practice was found to be less helpful, as it used contradictory language, sometimes suggesting such cases ‘must’ come to court, elsewhere indicating they ‘should’ as ‘a matter of practice’ ([19], para. 6.18, 8.18, 8.19). However, according to Lady Black, rulings succeeding the 2005 Act also did not convey any requirement ([11], para. 98).

The ECHR equally did not give rise to the need for such a requirement. UK law appeared fully compliant with the ECHR, since it has a compatible regulatory framework enshrined in the Act, Code and professional guidance (e.g. [20]), which requires clinicians to take into account the views of the patient, those close to him, and other professionals, and the court is always available whether or not there is a dispute. Lady Black noted that neither CANH nor these patients should be singled out for court oversight; CANH is medical treatment, so the general regulatory framework can apply.

Lady Black closed, however, with the suggestion that, if

the way forward is finely balanced, or there is a difference of medical opinion, or a lack of agreement to a proposed course of action from those with an interest in the patient’s welfare, a court application can and should be made ([11], para. 125).

As such, difficult cases can and should still come to court, but there is otherwise no requirement for a court to authorise the withdrawal of CANH from a patient with a PDOC.

### Welcome consequences?

With respect, the Supreme Court may have overstated its view that there was no requirement to bring these cases to court; everyone else – including some other judges – assumed there was [21, 22]. Certainly, the language oscillated between ‘should’ and ‘must’, but the latter undeniably featured, for example, in Lord Goff’s reference in *Bland* to there being a ‘requirement’ for such cases to come to court ([4], p. 874). Ironically, Lady Black’s judgment even threatens to re-open the can of worms that her ruling sought to close. There is some equivocation in her language regarding future cases that might merit a court ruling: she says there will be situations in which an application ‘can and should be made’, or ‘will be required (or desirable)’, and also that unresolved disputes ‘must inevitably be put before the court’ ([11], para. 109, 125, 126). This equivocation is unhelpful. Future situations are conceivable, in which clinicians and those close to the patient disagree about treatment withdrawal, but this wording threatens to leave neither group certain about the way forward. On one reading, a disagreement ‘must’ come to court; on another, a judicial decision may be ‘desirable’ but is not obligatory. Presumably, Lady Black’s comments should be read charitably in light of the overall judgment, which (inter alia) holds that the courts cannot and did not create an obligation as such. As such, Lady Black seems to be offering good practice recommendations: difficult cases should – but not must – come to court.

Despite this unwelcome new uncertainty, there are various reasons to welcome this ruling, many of which were noted by Lady Black. First, the decision brings English law in line with law elsewhere [23, 24]. Second, the decision restores some coherence to English law [24, 25]. The court’s appreciation of the legal and ethical questions arising in and from *Bland* was laudable, but the legality of removing CANH was settled long ago (see also [26]), and it seemed unusual to single out this treatment (CANH) and these patients (with PDOC) for judicial oversight, particularly in situations where every interested party was in agreement. Third, families will no longer routinely be required to go to court, with all the distress this can entail [24]. Fourth, the financial costs incurred by the health service, and even some families,^2^ will be saved; the legal process has been estimated to cost £122,000 per patient [28]. Finally, the ruling has the potential to reduce the delay – an additional 9 months on average – in getting the outcome that some families and clinicians want i.e. the withdrawal of CANH [28].

The ruling is therefore likely to have positive effects. But negative consequences are also conceivable, of which I will explore two. The first is the possibility that, following this decision, patients with PDOC might die too soon, but, secondly and conversely, there is also the possibility that such patients might live too long.

### Dying too soon?

The Kitzingers’ research indicates that families’ views change with experience and as prognoses become clearer [29]. Early months and years might be spent seeking treatment and hoping for recovery, but many later come to view their loved one as having suffered ‘a fate worse than death’ during this time [29]. Yet, this does not necessarily mean that every family welcomes the prospect of a death for their loved ones that is initiated via the removal of tube-feeding. For some, this was because the method (i.e. withdrawal of CANH), rather than the outcome (i.e. death), was considered ‘too cruel’ ([29], p. 159); the Kitzingers have, indeed, argued that there may be a role for active euthanasia here [30]. For other families, however, it was the outcome, regardless of the means by which it eventuated, which was problematic. One such wife describes her ‘battle’ to defend her husband’s life ([29], p. 158). Contrary to what such a spouse might wish, however, and despite the suggestion in the new ruling that cases of dispute should (or must?) still come to court, there are at least three reasons why the ruling in *Y* might increase the chances of life-sustaining treatment being withdrawn.

First, some consider the previous requirement for a judicial decision in all such cases to have been a deterrent to seeking the removal of CANH. Some of the Kitzingers’ participants were reportedly reassured by the existence of the perceived legal obstacle, one taking comfort from being informed by a lawyer about ‘how hard it is to turn off someone’s feed. […] you have to go to the High Court and it’s a very big thing to have done’ [31]. The ruling in *Y*, of course, removes this particular obstacle, and thus one clear deterrent to the removal of CANH. Where, however, the doctors wish to discontinue treatment, but the family wants it to continue, the family can continue to take comfort from these perceived legal hurdles, which might still serve to deter the clinicians from approaching the court.

Pro-treatment families might nevertheless be disquieted, secondly, by the possibility that cost considerations might exert some influence in the clinic. The courts have insisted that their focus is on the best interests of the individual patient, rather than any ‘external’ considerations, such as the costs of treatment ([32], para. 205, 206). These can be considerable, with the cost of sustaining a PVS patient estimated at £90,000 per year [33]. The courts may be wary of factoring this (overtly) into their decisions but, especially in an era of austerity and stretched healthcare resources, these considerations at least have the potential to influence decisions about (non-)treatment.

There is, however, a third reason why patients might die too soon, which could prove to be the most influential, since it focuses on the legal logic emerging from some of the cases to date, which will presumably (continue to?) guide clinical practice in this area. This legal logic strongly implies that CANH should – maybe even *must* – stop, at least if or when the patient is confirmed to be in a PVS. Estimates suggest that, since *Bland*, the courts have adjudicated on the fates of more than 100 patients in the PVS ([33], p. 12). It appears that every case has resulted in the withholding or withdrawing of CANH. The one apparent outlier case involved the court authorising a short trial of a sleeping pill, in an experimental final attempt to see if this could restore the patient’s consciousness [34]. It did not, and the matter returned to court, where the withdrawal of CANH – which the family had initially sought – was authorised [35]. The same result has uniformly obtained in other PVS cases, including those in which family members were opposed to withdrawal (e.g. [36]). Rightly or wrongly, the courts’ role in PVS cases essentially became one of confirming the diagnosis {24];^3^ once this matter was settled, the withdrawal of CANH would be authorised by the courts, seemingly regardless of the family’s views.

The test for making such a decision appeared – and appears – to be the best interests of the patient, a multi-factorial test that is now enshrined in the Mental Capacity Act 2005 ([7], section 4). That certainly continues to be the test applied to other incapacitated patients, including those in the MCS [38]. A best interests decision requires the courts (and, by extension, other decision-makers) to weigh up the different factors for and against treatment before reaching their decisions. However, the judicial approach to patients in the PVS has differed considerably. The courts have generally confirmed that the proper question is whether treatment, as opposed to non-treatment, is in the best interests of the incapacitated patient ([39], para. 20). However, the courts have consistently answered that question in the negative if the patient is in a PVS and they have held that, in these cases, there is no question of balancing different considerations, such as what the patient and/or his or her family might want. As such, the courts do not undertake a balancing exercise in these cases: once the PVS diagnosis is confirmed, treatment will be withdrawn.

These negative answers arise from the legal logic deployed by the judges. The rulings have suggested that continued treatment of those in the PVS is ‘futile’ ([4], p. 869 [40];, para. 66). The position originated in *Bland*, in which Lord Mustill also stated that the patient had ‘no best interests of any kind’ ([4], p. 897). Although the 2005 Act supersedes *Bland*, and the courts’ understanding of ‘futility’ is evolving [41], both elements of this logic have endured, with Newton J in *Re P* [2015] stating succinctly:

If P is in VS, and there is no real prospect of recovery, then it would not be in his best interests to continue treat him (because a person in a continuing vegetative state has no interests and therefore no best interests) ([42], para. 13).

This logic has been challenged. Keown, a defender of the sanctity (or intrinsic value) of life, has argued that CANH is not futile, because it is evidently feeding the patient, and countered that it would certainly be contrary to a PVS patient’s interests to use him or her as a sideboard ([43], p. 494). Baines is more sympathetic to Lord Mustill’s position, although he concedes that using patients like furniture might still be wrong, albeit on some other basis, such as an affront to the patient’s dignity [44]. But, whatever its philosophical merits, the legal logic remains: the PVS patient has no best interests and treatment is futile. Clinicians presumably must operate in the shadow of the courts. If so, the message to them appears to be clear: once the PVS diagnosis is settled, CANH can and should be stopped.

This legal message might amount to more than a recommendation: it might be considered a mandatory requirement. Some of the Law Lords in *Bland* held that there was no *duty* to treat a PVS patient if treatment was not in his or her best interests ([4], pp. 858, 877). Yet, some went further, holding that there was no *entitlement* to treat in such a situation – indeed, doing so could amount to trespass or assault ([4], pp. 883, 885, 876, 877). The Supreme Court has since repeated the latter proposition:

If the treatment is not in his best interests, the court will not be able to give its consent on his behalf and it will follow that it will be lawful to withhold or withdraw it. Indeed, it will follow that it will not be lawful to give it ([39], para. 22)*.*

If that logic must move from the courtroom to the clinic, then clinicians apparently *must* cease treatment once an irrecoverable PVS diagnosis has been confirmed. It warrants emphasis that this is the *legal* logic, which (as we will explore in the next section) might not be shared by clinicians. But if, regardless of any misgivings they might have, clinicians must now absorb and apply the legal logic, then treatment must apparently be withdrawn from those in the PVS. There is also reason to suspect that, in practice, this requirement could apply not only to patients in the PVS, but also to those in the MCS. Diagnosis of PDOCs requires specialist expertise and is notoriously difficult, with the Kitzingers describing a ‘diagnostic illusory’ [45], and some experts arguing that ‘It is not possible to identify the vegetative state as an obviously different state from the (lower end of) minimally conscious state’ ([46], p. 443). If that is the case, then it is conceivable that not only patients in the PVS, but also those in the MCS will face the prospect of dying too soon.

In sum, the removal of a perceived deterrent, the vexed question of costs, and (especially) the legal logic in favour of withdrawal might therefore increase the likelihood of treatment routinely being withdrawn from patients in PDOC. The prospect of these (or perhaps any) patients dying too soon for lack of sufficient safeguards is likely to concern many, and not only those family members or others who believe in the sanctity of human life. Series, for example, worries that the ruling in *Y* ‘has more deeply entrenched a very wide ranging *de facto* power to make decisions with serious human rights implications, and very limited safeguards’ [47]. If treatment withdrawal does increase or become the default position, then this might have further unintended consequences. For example, routine withdrawal of treatment from these patients could inhibit future research that might benefit current and future patients. As the courts have previously acknowledged, there may be situations in which therapeutic research (or an experimental procedure) has the potential to benefit the individual patient [34]. Research might also come to benefit the whole cohort of patients. Yet, if the numbers of such patients who are sustained declines substantially (potentially even to zero), then there may be too few (or no) such patients to enable – or event warrant – research in this area. However, we need not labour these potential consequences, because there is reason to suspect that the more likely consequence will drive in the opposite direction: rather than patients dying too soon, they might be more likely to live too long.

### Living too long?

A range of factors, which cluster around the patients’ families, the clinicians, and the health system, suggests that a more likely consequence of this ruling is that treatment will continue for patients in PDOC – whether or not this is best for the particular patient.

First, *patients’ families* may be one of the (unwilling or unwitting) barriers to non-treatment. The Kitzingers’ research indicates at least three reasons why this might prove to be the case. First, families might lack the pertinent clinical and legal knowledge. Families tend to depend on clinicians for information about their loved one’s condition, prognosis and the (treatment and non-treatment) options that are available. The Kitzingers’ research suggests, however, that there are persistent failures in communication. One family reported such a lack of communication from the clinicians, which left them ‘second guessing at the future, at the state of Mum, where she was going’ ([31], p. 13). This family, like others, reported ‘not even knowing’, ‘years down the line’ that withdrawal of treatment was an option ([31], p. 13).

This is significant because, second, the Kitzingers have found that families, rather than clinicians, tend to be the ones who propose or lobby for the withdrawal of CANH. For example, one of the Kitzingers’ articles focuses on the experiences of five families and, ‘[i]n all five cases, it was family members (not healthcare teams) who initiated discussion about withdrawal of ANH’ ([31], p. 13). These families were also ‘active agents in the court application process’, who ‘saw themselves as behaving courageously on behalf of their relative in actively supporting the decision to withdraw ANH’ ([31], p. 12). The family therefore needs the requisite knowledge and a measure of courage to push for withdrawal; where these are lacking, treatment seems likely to continue.

Third, some of those families who have gone to court took comfort from the sense that the court was sharing with them the burden of deciding. Halliday et al. describe the judgments as containing ‘therapeutic jurisprudence’, since they ‘operate as memorials of the living dead’ and formally affirm the suffering the family has endured ([24], p. 569). The court, of course, is ultimately the one to decide and research participants have spoken of the court having ‘shielded’ them from assuming sole responsibility for the decision to end treatment ([31], p. 13). In the absence of the court’s shield, however, it is conceivable that families may consider such a (perceived) responsibility to be too great a burden, so treatment will continue.

These findings combine to suggest that families might lack the knowledge, energy or desire to seek treatment withdrawal, so treatment will continue by default. The *clinicians* also play an important role in compelling this result. Research points to at least five reasons why treatment might continue.

First, there have long been concerns about over-treatment in healthcare generally. According to a recent medical editorial:

Ineffective and harmful medical practices have always been with us, but the scale and institutionalisation of overdiagnosis and overtreatment have expanded exponentially in the last few decades ([48], p. 116).

There may be many reasons for over-treatment but one to which the Kitzingers refer in this specific context, is that of the conscientious beliefs of the treating clinicians. Some families to whom they spoke believed that proceedings were deliberately obstructed or prolonged, because the clinicians had ethical or religious objections to treatment-withdrawal [33, 49]. These sorts of objections will undoubtedly persist and they might similarly prevent families from seeking or securing the withdrawal of treatment without going to court.

Moreover, third, empirical research reveals that clinicians lack adequate knowledge about the complexities of PDOC. As we have seen, diagnosis is complicated, requiring specialist input, using specialist tools. Official figures are lacking, but estimates suggest that, in the UK, there are 4000–16,000 patients in PVS, with three times as many in the MCS [1]. These are substantial numbers, but many healthcare professionals still lack experience in dealing with such patients ([31], p. 13). This means that the professionals can be ignorant of good practice in this area. For example, the Kitzingers report on a case, in which the team informed the family that a PVS diagnosis could only be confirmed 1 year after the precipitating event ([33], p. 3). This is accurate, but only in cases where that event was a ‘traumatic’ injury ([2], p. 10); in the case in question, the cause was a non-traumatic injury, so the diagnosis could have been confirmed after only 6 months.^4^

Fourth, as the latter case demonstrated, there can be delays in gathering the requisite information, which can even mean that the opportunity to allow the patient to die is lost. In the aforementioned case, some of the necessary diagnostic tests only occurred 2 years after the patient’s injury, ‘in spite of there being no clinical reason for the delay and in spite of the family pressing for such assessments to take place as soon as possible’ ([33], p. 3). Other cases have involved even longer delays, with one PVS patient being sustained for 23 years [50]. Delays are problematic, say the Kitzingers, in part because the ‘window of opportunity’ for allowing death, through the withholding or withdrawing of treatment, can close [49]. This tragically appears to have been the case for their sister, Polly, who, after 2 years in a PDOC following a car accident, regained consciousness ‘but her brain injuries have left her permanently dependent on 24/7 care for all her needs’ [51]. Although her sisters are convinced that Polly would not value life in this condition, the doctors had declined to withdraw feeding and, now, the opportunity to do so has gone, since Polly receives nutrition orally.^5^

An inclination to over-treat, delays and ignorance of PDOC are not the only drivers of (continued) treatment, however. Fifth, there is evidence that clinicians are also ignorant of the law in this area, with its central focus on the best interests of the patient. In 2014, the House of Lords’ Post-Legislative Scrutiny of the Mental Capacity Act found that the legislation ‘needs a higher profile among professionals in order to be properly understood and effectively implemented’ ([52], para. 135). Little wonder that families are legally ill informed if clinicians are too. The Kitzingers accordingly express concern about ‘the (sometimes total) absence of best interest consultations, default presumptions of continued treatment and decision making based on criteria other than the best interests of the patient’ ([31], p. 16).

Families and clinicians may therefore act, whether intentionally or not, as barriers to treatment withdrawal. But so too does the *health system* in which they all find themselves, due to a confluence of factors [50]. The system is fragmented, disjointed and ineffective, and it lacks the resilience to respond to emerging dilemmas. Health professionals administer the system, but they do not fully understand it and lack the support to navigate it. There is a lack of access to medical and legal expertise. And, as we have already seen, there are failures in communication, not only between families and clinicians, but also between different professionals [51]. These factors lead the Kitzingers to conclude that ‘the institutionalized provision of long-term treatment-by-default normalizes sustaining life in PVS making it difficult for families (or staff) to consider alternatives’ ([51], p. 13).

### Going forward?

The ruling in *Y* has therefore removed one barrier to the withdrawal of CANH from patients in PDOC, which is likely to have some positive effects. It might, however, either tend to patients dying too soon or else lead to them living too long. Either looks like a plausible possibility. The possibility that patients might die too soon appears the less likely – but is still conceivable. The legal logic, which holds that PVS patients (at least) should not be treated because they lack interests and treatment is futile, appears to be the strongest driver in this direction. Admittedly, that logic appears not – or not yet – to have moved from the courtroom to the clinic. However, that might be because the logic could not hitherto have moved between settings, since a court decision was required (or, at least, recommended). Now, of course, there is no such uniform barrier, so the logic might well transfer to the clinic, resulting in many more than the 100 or so patients to date having CANH withdrawn. Judging by the Kitzingers’ research, however, the second possibility of patients living too long appears to be the more likely outcome, since – whether intentionally or not – patients’ families, clinicians, and the health system combine to promote treatment-by-default.

Which, if either, of these is a desirable or appropriate outcome is debatable. The ethical defensibility of discontinuing treatment from these, and other, patients obviously remains contested. There is, however, a case for favouring a pluralistic, case-by-case approach, in which decisions are based on the particularities of the patient. Such a case finds support both in practice and in principle.

In practice, the law (and professional guidance) generally adopts a principle – the best interests principle – which accommodates a wide variety of factors and supports a wide variety of decisions. Although there are pockets of uniformity, and therefore groups of cases in which a ‘rule’ appears to operate, a decision for or against treatment in a particular case is not generally a foregone conclusion [53]. This pluralism can be a source of strength and of weakness ([54], p. 173). On one view, the concept of law requires there to be rules, which can guide human behaviour ([55], p. 96). However, the law on best interests generally appears to vest a great deal of discretion in decision-makers, such as judges [56]. Plurality and discretion can create uncertainty and unpredictability, and accordingly inhibit the law from meeting its action-guiding goal [38, 53].

Yet, the flexibility of the legal standard can also be judged more positively. Rather than ‘elaborate’ a ‘one-size-fits-all’ approach, which might be tethered to a particular value, the current approach merely ‘enumerates’ some of the various different factors and values that can have a bearing on the case at hand [57]. Such a position is defensible in principle: value pluralism is tolerant of moral diversity, since it recognises that there can be a plurality of moral values, which are not necessarily amenable to ranking or resolution [58]. Value pluralism also recognises that investing wholly in one value and therefore in one decision can be risky, since it may prove to be the wrong value and wrong decision. Applied to our cases, it may be right for *some* patients with PDOC to live and *others* to die. What value pluralism encourages, however, is recognition that the ‘right’ decision can vary between patients.

This defence is admittedly brief, but it at least begins to provide a basis for holding to a pluralistic standard. There are, of course, alternatives to the best interests standard, such as a substituted judgment standard, a harm standard or an approach which is more oriented towards what incapacitated persons do or would want [59–61]. The Law Commission has favoured the latter approach, proposing that decision-makers should ‘be required to consider the person’s ascertained wishes and feelings when a best interests determination is being made’ ([62], para. 1.36), although this proposal did not feature in the Mental Capacity (Amendment) Act 2019, which reformed the law governing deprivation of liberty [63]. Whether or not proposals like these will gain ground, the latter autonomy-led arguments do at least provide further support for the proposition that decisions should be made on a case-by-case basis, which involves looking to the particularities of this patient.

Bringing these arguments together, the first recommendation is that law (and, correspondingly, professional guidance and practice) should continue to adopt a pluralistic standard, according to which decisions should be made on the basis of the plural factors relevant to the case in question. Although more research is needed here, [64] the best interests standard might offer a suitable basis for making decisions in this area (and, indeed, others). If this standard, or some version thereof, is retained, then more certain and predictable rules might yet emerge from its application, but – importantly – there should still be careful attention to what is appropriate for *this* patient in *these* particular circumstances. Hopefully, through such an approach, law can serve to guide people, whilst also respecting diversity.

Whether or not the best interests standard is for the best, this is undoubtedly the standard that the law currently adopts. Both the Kitzingers and the House of Lords’ 2014 review of the Act implicitly recognise that the existing law already has the capacity to address many of the perceived problems we have surveyed. It follows that the problems of dying too soon or living too long can be avoided or ameliorated, if effort is expended in ensuring that people are aware of the law and they practice accordingly.

First, then, the relevant people should be made aware of the law in this area. This will require effort on the part of healthcare professionals, the health service, and perhaps also patients and families themselves. The health service should at least ensure that professionals are suitably informed and educated about the Mental Capacity Act, its Code and applicable professional guidance, so that professionals can, in turn, inform and support patients and their loved ones. The law has long been in place but, as the 2014 review and the Kitzingers’ research found, professionals appear not to be adequately informed. Important headlines contained within the legal framework that must be grasped include the focus on the best interests of the patient, which requires reference to his or her known or anticipated wishes and consultation with his or her loved ones, plus those provisions pertaining to surrogate decision-makers and advance decisions to refuse treatment.

Second, and focusing specifically on best interests decisions, robust processes are needed, to ensure that decisions are timely, regularly reviewed, take due account of the relevant factors and perspectives, and are appropriately documented. Best interests decisions should certainly be more timely than in some of the cases we have considered, especially if it will be best for some patients to slip through the ‘window of opportunity’ i.e. have treatment withdrawn. Review – including, where necessary, recourse to second opinions – is also important, to ensure the decisions are indeed for the best at the relevant time. Assessing what is best will require attention to all aspects of the patient’s treatment and reference to a plurality of factors and perspectives, including not only family views, but also ‘high-quality’ diagnostic and prognostic information ([50], p. 138). Here, as elsewhere in healthcare, good communication will be essential, both between professionals and between professionals and families.

In this regard, it is also important to clarify where responsibility for the decision lies and why the family’s views are being sought. Put simply, the decision lies with the responsible clinician – although, admittedly, *who* this is will sometimes need to be clarified ([50], p. 140). As such, the family does not make the ultimate decision; rather, its views will be sought in order to ascertain what the patient would have wanted. Families therefore do not carry the responsibility for decisions, although they should be involved in making them. It also follows that, unlike some of those cases considered here, families should not be required to raise the prospect of withdrawal. Finally in this regard, the Kitzingers recommend that organisations responsible for inspecting care (such as the Care Quality Commission) and for funding care (such as Clinical Commissioning Groups) should ensure that appropriate decisions are being made, by seeking suitable documentation and holding decision-makers to account ([50], p. 139). The documentation should include records of best interests decisions, and it may also be useful to create a register of all those entering (or in) a PDOC ([50], p. 140 [65];, p. 340).

Hopefully, by acting on these sorts of recommendations, we can prevent families – and some professionals – from feeling ‘trapped in a system of “care delivery” which seems to have its own logic and momentum’ ([50], p. 137). The Kitzingers were here referring to the prospect of treatment-by-default, but it is also important to avoid withdrawal-by-default. Rather than generalised propositions, which point automatically to one or other consequence, regardless of who the patient is, individualised decisions are needed, which seek to secure the right result for the particular patient.

Fortunately, new professional guidance captures many of these suggestions. In December 2018, the British Medical Association and Royal College of Physicians published guidance, endorsed by the General Medical Council, on CANH for patients who lack the capacity to consent [3]. Just as the law, following *Y*, no longer singles out those with PDOC, the guidance covers not only these patients, but also those with progressive neurodegenerative conditions, multiple comorbidities or frailty, or other brain injuries. Amongst the topics covered in the guidance are: the legal position; who makes decisions and who must be consulted; conscientious objections; clinical assessments and second opinions; best interests assessments; managing disagreement and uncertainty; keeping records; and governance and audit.

The guidance addresses many of the points made here.^6^ The opening summary acknowledges that errors might be made in either direction, since a ‘wrong decision … could result either in CANH being withdrawn too soon – thus depriving the patient of an opportunity to live a life they would value – or of it being continued too long – forcing the individual to continue a life they would not have wanted’ ([3], p. 8). The guidance also repeatedly makes explicit the importance of an individualised, case-by-case approach, which is focused on the patient:

The central point to keep in mind, throughout the decision-making process, is that the decision is about what is in the best interests of the *individual* patient, not what is best for those who are close to them, what most people in their situation would want, or what is best for the family, the care team or the providers or funders of care ([3], p. 30).

The Kitzingers participated in the drafting, so it is not surprising that (on three occasions) the guidance counsels against the continuation of treatment ‘by default’ ([3], pp. 23, 26, 71). In line with recent court rulings, and indeed arguments advanced elsewhere, the guidance is also notably orientated towards an autonomy-driven reading of ‘best interests’ [39, 61]. In the opening summary, for example, the point is made that the presumption in favour of providing life-sustaining treatment ‘can be rebutted if there is clear evidence that a patient would not want CANH provided in the circumstances that have arisen’. ([3], p. 6) The guidance also emphasises the need for robust best interests processes, along with suitable record-keeping and monitoring (e.g. [3], pp. 56–68). Finally, the guidance explicitly notes the need for training and support in the implementation of the guidance (and, by extension, the law) ([3], pp. 71–74), with the website including training materials (https://www.bma.org.uk/advice/employment/ethics/mental-capacity/clinically-assisted-nutrition-and-hydration).

There is, then, much to welcome in this guidance. However, its length might inhibit its uptake, since the main guidance for doctors runs to 100 pages. This does at least ensure that the requisite detail and the associated complexities are conveyed. Moreover, the main guidance is supplemented by, for example, a summary and infographics, which should help to ensure that the key messages are accessible (https://www.bma.org.uk/advice/employment/ethics/mental-capacity/clinically-assisted-nutrition-and-hydration). The messages are also, commendably, not only directed towards doctors, as there is also information targeted to families and to other healthcare professionals, funders and managers. Of course, as experience with the Liverpool Care Pathway suggested, efforts will now be needed to ensure that the relevant messages are heard and heeded in practice [13]. In short, attention must now turn to translating the guidance into practice, by ensuring that those involved in these decisions – and particularly the responsible doctors – are suitably trained and supported going forward.

## Conclusion

Questions continue to surround the treatment and non-treatment of patients with prolonged disorders of consciousness, including some about the language that should be used to describe and categorise these patients [66, 67]. The Supreme Court has decisively answered one such question, finding that there is no general requirement for decisions about the (non-)provision of clinically assisted nutrition and hydration to be made by a court. This appears to be a welcome decision, since it enhances the coherence of the law and avoids or reduces the emotional and financial costs associated with recourse to the courts.

The ruling might nevertheless have (unintended) negative consequences. Patients might be at risk of dying too soon, particularly as the courts’ logic to date seems inclined towards withdrawal-by-default if the patient is in a PVS. Alternatively, patients with PDOC might be at risk of living too long, since there are various individual and systemic factors which seem to promote treatment-by-default. Blanket policies could be avoided if clinicians (and others) are better informed about the law and they practice accordingly. Hopefully, recent professional guidance and its accompanying training materials will help to clarify matters for those involved – provided, of course, that sufficient effort is put into delivering training and support, so that the relevant messages can be heard and heeded. Ethically, as the guidance also emphasises, it appears that a patient-specific approach is more defensible than generalised, objective judgments. Quite what the basis for these decisions should be, however, remains open to question. The best interests standard can accommodate and encourage a patient-specific approach, but its ethical defensibility has been challenged and alternatives have been proffered. For its part, the guidance follows the courts in emphasising the need to look to what this patient would (or would not) want in ascertaining his or her best interests. Future research will hopefully shed more light on how that standard might best be interpreted for these – and other – patients and whether it should be modified or even replaced entirely.

## Data Availability

Not applicable.

## Abbreviations

CANH: Clinically-assisted nutrition and hydration
ECHR: European Convention on Human Rights
MCS: Minimally conscious state
PDOC: Prolonged disorder(s) of consciousness
PVS: Permanent vegetative state
VS: Vegetative state

## Footnotes

1 The arguments herein essentially pick up the Kitzingers’ gauntlet, by (in part) using their empirical findings to inform the following ethical analysis. Questions might arise about the legitimacy of these methodological manoeuvres, although they may be defended by reference to developments in ‘empirical bioethics’ research (e.g. [17]).

2 The courts have noted ‘alarming’ situations in which legal aid was unavailable to the families bringing or participating in proceedings ([27], para. 12). New guidance from the British Medical Association, discussed below, proposes that court ‘proceedings should be initiated and funded by the relevant NHS body responsible for commissioning or providing the patient’s treatment’ ([3], p. 40).

3 Although, commenting on *Y* ([11], para. 119), see also [37].

4 In this regard, note that Mr. Y had suffered cardiac arrest and lost consciousness in June 2017. He was diagnosed in a vegetative state in October and his case came to court on 1 November 2017 ([11], para. 3, 4). The Royal College of Physicians guidance (ibid) states that ‘VS may be classified as “permanent VS” if it has persisted for … >6 months following anoxic or other metabolic brain injury’ ([2], p. 10). As such, at the time of the initial hearing, Mr. Y was not (yet) in a *permanent* vegetative state.

5 Her sister reports, however, that ‘we have a “do not attempt resuscitation” notice informed by her values and beliefs and she wouldn’t be treated for life-threatening infections’ [51].

6 Please note that the author provided comments on drafts of the guidance.
